# Acute abdominal pain presenting as a rare appendiceal duplication: a case report

**DOI:** 10.1186/1752-1947-6-79

**Published:** 2012-03-08

**Authors:** Ali Mahmood, Nadia F Mahmood, Jennifer L Williams

**Affiliations:** 1 Clinical Faculty, Department of Surgery, Minimally Invasive Surgery Consortium, University of Texas Medical School at Houston, 16605 SW Freeway, Suite 430, Sugar Land, TX 77479, USA; 2 Baylor College of Medicine, Department of Radiology, Texas Children's Hospital, 6621 Fannin Street MC2-2521, Houston, TX 77030, USA

## Abstract

**Introduction:**

Appendiceal duplication is a rare anomaly that can manifest as right lower quadrant pain. There are several variations described for this condition. We recommend aggressive operative management should this anatomical variation present in the presence of acute appendicitis.

**Case presentation:**

We report the case of a 15-year-old African American girl who presented to our hospital with right lower quadrant pain and was subsequently found to have appendiceal duplication.

**Conclusion:**

There are two categorical systems that have described and stratified appendiceal duplication. Both classification systems have been outlined and referenced in this case report. A computed tomography scan has been included to provide a visual aid to help identify true vermiform appendiceal duplication. The presence of this anatomical abnormality is not a reason for surgical intervention; however, should this be found in the setting of acute appendicitis, aggressive resection of both appendices is mandatory.

## Introduction

Appendiceal duplication is a rare anomaly that has been described less than 200 times in the literature. The incidence of duplicated appendices has been previously reported to be approximately 0.0004% [1]. This anatomical finding has been associated with intestinal, bone and genitourinary abnormalities as well [2-5]. While the presence of appendiceal duplication in the absence of inflammation is not always and/or immediately a surgical issue, once there is evidence of appendicitis, prompt and aggressive surgical intervention is necessary.

## Case presentation

A 15-year-old African American girl presented to our emergency room with abdominal pain. The pain had started 48 hours previously, with the onset in the supra-umbilical region and subsequent radiation to her right lower quadrant. The pain was exacerbated by movement of her right lower extremity. Our patient denied nausea, vomiting, chills or rigor. Upon physical examination, she had point tenderness in her right lower quadrant, without rebound tenderness, guarding or rigidity. Her white blood cell count was mildly elevated at 11,000 k/CMM (cubic millimeter) without leukemoid shift. A urine analysis did not reveal any abnormalities and a urine pregnancy test was negative.

**Figure 1:**
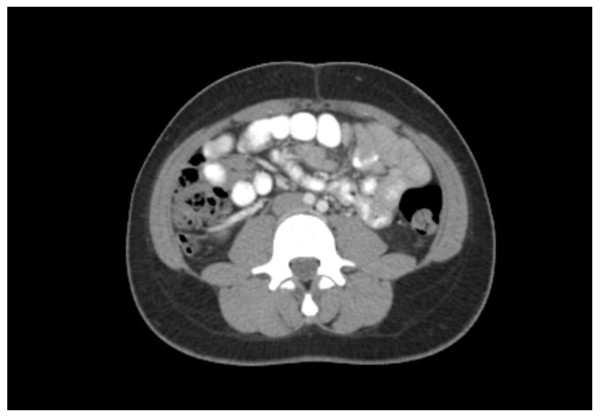
**Axial computed tomography images depicting the appendiceal duplication**.

**Figure 2:**
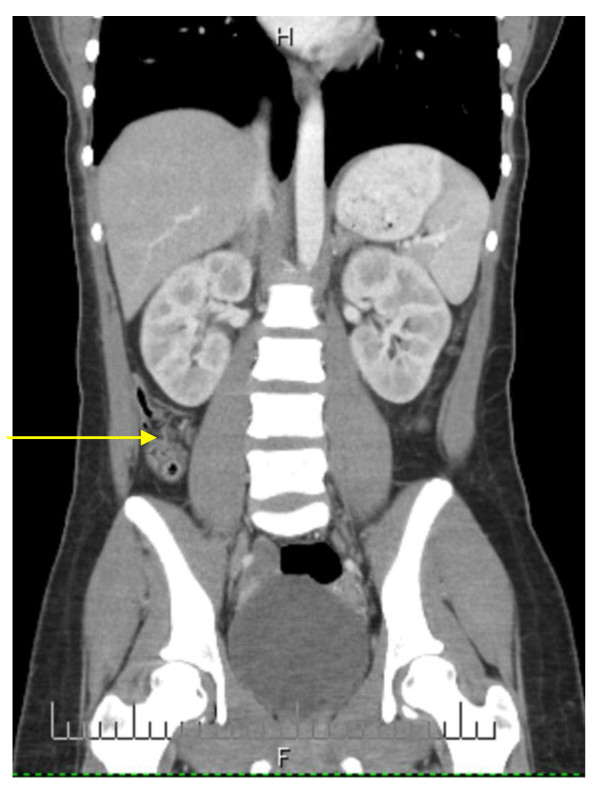
**Coronal computed tomography images depicting the appendiceal duplication (arrows)**.

An ultrasound was performed, which did not visualize the appendix or any inflammatory changes. A computed tomography scan showed a retrocecal appendix (Figures 1 and 2). The lumen of her appendix bifurcated 2 cm distal from its cecal origin, without inflammatory changes.

Our patient was admitted and started on intravenous fluids accompanied with bowel rest. She responded to medical management and was eventually discharged home.

## Discussion

There have been two classification systems proposed to categorize appendiceal duplication. The first classification system was described by Waugh in 1941 and consisted of three categories [6]. The first category described appendiceal duplication where both appendices originated from one cecum. The appendiceal lumens were juxtaposed to each other, with submucosal fibrous communication arising at various lengths from the cecal base. The second type of duplication was depicted by two appendices, located on two distinct and polar sides of the ileocecal valve. The last category described one appendix arising from the normal anatomical point with a second appendix originating from a distal point along the tenia.

A second system to categorize appendiceal duplication was introduced in 1963 by Wallbridge, which interestingly also consisted of three classifications [7]. Type A described two appendices arising from one cecum, with one appendix smaller and shorter than its counterpart, appropriately classified as a partial appendix. Type B described two complete appendices, each stemming from a single cecum. This category was further subdivided into B1 and B2. B1 depicted the two appendices arising from either side of the cecum, approximately 180 degrees apart, at a fixed anatomical point. B2 described one appendix arising from the cecum and the second appendix originating from the tenia, distal from the cecum. Type C was used to categorize two appendices, along with two cecums, one appendix arising from each respective cecum, although this category is exceedingly rare [8,9].

It is important to distinguish appendiceal duplication from other differential diagnoses of bowel etiology. Ultrasound is often used to diagnose or visualize the appendix; however, should the ultrasound be negative and the index of suspicion remain high, for acute appendicitis, we recommend computed tomography of the abdomen and pelvis with oral and intravenous contrast. Barium enemas have been used to diagnose appendiceal duplication; however, we do not recommend this-particularly as there is a risk of perforation in the presence of potential appendiceal inflammation and subsequent complicated peritonitis [10]. The diagnosis of appendiceal duplication can be confirmed with pathological and histological examination. The presence of lymphoid tissue within the wall of the appendix does differentiate it from bowel diverticulum [11]. Although the disease is associated with several abnormalities, it is not pathognomonic to a specific disorder. Skeletal surveys, bone biopsies or investigation of the genitourinary system do not supercede prompt evaluation of the appendix.

Management of acute appendicitis, in the clinical scenario of appendiceal duplication, warrants complete appendectomy. Obviously this has to be performed for Waugh type1 duplication and for Wallbridge (later renamed Cave-Wallbridge) Type A; however, we advocate this for all symptomatic cases of acute appendicitis with appendiceal duplication, irrespective of only single appendiceal inflammation. Our patient did not have any evidence of inflammatory changes; hence she was discharged without operative intervention with a working diagnosis of bowel gas pain versus gynecological physiologic pain.

Appendiceal duplication found incidentally when operating for other abdominal pathology does not immediately warrant a complete appendectomy. In the setting of an acute inflammatory disease, such as Crohn's disease, an appendectomy should not be performed.

## Conclusion

Appendiceal duplication is a rare anomaly that can present in children. The chief concern is to address the inflammatory changes involving the appendix and, should appendicitis be suspected, removal of both appendices should be performed. A previous single appendectomy could potentially lead to confusion, delay or potential compromise in patient care.

## Consent

Written informed consent was obtained from the patient's legal guardian for publication of this manuscript and any accompanying images. A copy of the written consent is available for review by the Editor-in-Chief of this journal.

## Competing interests

The authors declare that they have no competing interests.

## Authors' contributions

AM was a major contributor in writing the manuscript. NFM was a major contributor in evaluating the radiological aspect of the manuscript. JLW was a major contributor in editing and revising the manuscript. All authors read and reviewed the final manuscript.
